# Standardized training in the rating of the six-item positive and negative syndrome scale (PANSS-6)

**DOI:** 10.1192/j.eurpsy.2021.1580

**Published:** 2021-08-13

**Authors:** P. Kølbæk, D. Dines, J. Hansen, M. Opler, C. Correll, O. Mors, S. Østergaard

**Affiliations:** 1 Department Of Clinical Medicine, Aarhus University, Aarhus, Denmark; 2 Department Of Affective Disorders, Aarhus University Hospital - Psychiatry, Aarhus, Denmark; 3 Psychosis Research Unit, Aarhus University Hospital - Psychiatry, Aarhus N, Denmark; 4 Office Of The Executive Director, PANSS Institute, New York City, United States of America; 5 Department Of Psychiatry, New York University School of Medicine, New York, United States of America; 6 Medavante-prophase Inc, MedAvante-ProPhase Inc, New York, United States of America; 7 Department Of Psychiatry, The Zucker Hillside Hospital, Glen Oaks, New York, United States of America; 8 Department Of Child And Adolescent Psychiatry, Psychosomatic Medicine And Psychotherapy, Charité University Medical Center, Berlin, Charite Berlin, Padua, Germany; 9 Department Of Psychiatry And Molecular Medicine, Donald and Barbara Zucker School of Medicine at Hofstra/ Northwell School of Medicine, Aarhus, Denmark

**Keywords:** schizophrénia, psychopathology, rater training, PANSS

## Abstract

**Introduction:**

The six-item Positive And Negative Syndrome Scale (PANSS-6) is short psychometric valid scale quantifying the severity of core schizophrenia symptoms. Using PANSS-6 to guide treatment decision-making requires that staff members’ ratings are valid and reliable.

**Objectives:**

The objective of the study was to evaluate whether such valid and reliable PANSS-6 ratings can be obtained through a video-based training program.

**Methods:**

One-hundred-and-four staff members from Aarhus University Hospital - Psychiatry, Denmark participated in the training. Participants conducted baseline PANSS-6 ratings based on a video of a patient being interviewed using the Simplified Positive And Negative Symptoms interview (SNAPSI). Subsequently, a theoretical introduction video was displayed followed by five SNAPSI patient interviews. After each SNAPSI video, individual ratings were performed before a video providing the gold standard scores was displayed. The validity of ratings was estimated by calculating the proportion of participants not deviating from the gold standard scores with >2 points on individual items or >6 points on the PANSS-6 total score. Reliability was evaluated after each step in the training by means of Gwet’s Agreement Coefficient (Gwet).

**Results:**

By the end of the training, 72% of the participants rated within the acceptable deviations of the gold standard, ranging from 60% (nurses) to 91% (medical doctors/psychologists). The reliability improved (Gwet baseline vs. endpoint) for all PANSS-6 items, except for Blunted affect.

**Conclusions:**

The majority of the staff members conducted valid PANSS-6 ratings after a brief standardized training program, supporting the implementation of PANSS-6 in clinical settings to facilitate measurement-based care.

**Conflict of interest:**

Dr. Opler is a full-time employee of MedAvante-ProPhase Inc. Dr. Correll has been a consultant and/or advisor to or have received honoraria from: Acadia, Alkermes, Allergan, Angelini, Axsome, Gedeon Richter, Gerson Lehrman Group, Indivior, IntraCellular T

